# Visceral Perforation Following Hemostatic Powder TC-325 Application in a Patient With Gastric Cancer: A Case Report

**DOI:** 10.7759/cureus.83611

**Published:** 2025-05-06

**Authors:** Konstantinos Papantoniou, Theodora Kafentzi, Christos Sotiropoulos, Konstantinos C Thomopoulos, Christos Konstantakis

**Affiliations:** 1 Department of Internal Medicine, Division of Gastroenterology, Patras Medical School, University of Patras, Patras, GRC

**Keywords:** bleeding, endoscopy, hemostasis, malignancy, perforation, tc-325

## Abstract

Malignant tumors of the upper gastrointestinal (GI) tract are a common cause of GI bleeding, often presenting therapeutic challenges due to diffuse hemorrhage and poor visualization. Local hemostatic agents, such as TC-325 (Hemospray; Winston-Salem, NC: Cook Medical), have emerged as effective bridging therapies prior to definitive treatment. However, visceral perforation, though rare, is a serious complication associated with TC-325 use. We present a case of an 87-year-old male with metastatic gastric adenocarcinoma who experienced visceral perforation following TC-325 application for recurrent upper GI bleeding. Despite achieving initial hemostasis, the patient succumbed to complications of perforation. This study highlights the need for caution when using TC-325 in patients with advanced malignancies and friable tissue.

## Introduction

Non-variceal upper gastrointestinal bleeding (NVUGIB) is a significant cause of morbidity and mortality worldwide. While malignancies of the upper GI tract are a less common etiology, they pose unique challenges due to diffuse bleeding surfaces, friable tissue, and poor endoscopic visualization [1]. Topical hemostatic agents, such as TC-325 (Hemospray; Winston-Salem, NC: Cook Medical), have emerged as effective alternatives to standard endoscopic therapies, particularly in cases of malignant NVUGIB [2]. Although TC-325 is generally considered safe, rare but serious complications, including visceral perforation, have been reported [3,4]. Here, we present a case of visceral perforation following TC-325 application in an 87-year-old male with metastatic gastric adenocarcinoma, underscoring the need for caution in patients with advanced malignancies.

This article was previously presented as an e-poster at the 44th Panhellenic Congress of Gastroenterology on November 28, 2024, and at the Annual European Society of Gastrointestinal Endoscopy (ESGE) Days Congress on April 3, 2025.

## Case presentation

An 87-year-old male with a history of arterial hypertension, dyslipidemia, and chronic obstructive pulmonary disease presented to our emergency department with hematemesis, hematochezia, and hemodynamic instability. Laboratory tests revealed severe anemia with hemoglobin 4.9 g/dL (reference range: 11.8-17 g/dL). The patient had been previously diagnosed with metastatic gastric adenocarcinoma (linitis plastica), with secondary lesions in the liver and lungs. He had previously undergone multiple hospitalizations, both for recurrent upper gastrointestinal (GI) bleeding and transfusion-dependent anemia attributed to the tumor and not amenable to traditional endoscopic treatment. During a prior hospitalization, active bleeding from the gastric mass was successfully controlled with the application of TC-325 hemostatic powder (Hemospray) without complications.

**Figure 1:**
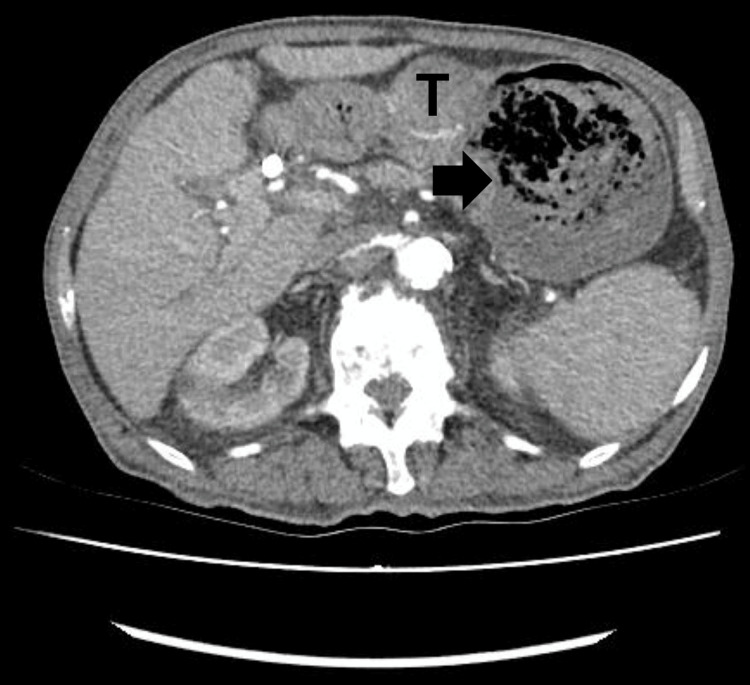
Gastric tumor (T) and intragastric blood (arrow) view at the time of patient admission.

At the current presentation, computed tomography (CT) angiography confirmed the gastric tumor as the source of bleeding, without evidence of active contrast extravasation (Figure 1). Given the patient’s hemodynamic status and the need for urgent intervention, endoscopic hemostasis with TC-325 was chosen due to its previous success and the limitations of alternative therapies.

**Figure 2:**
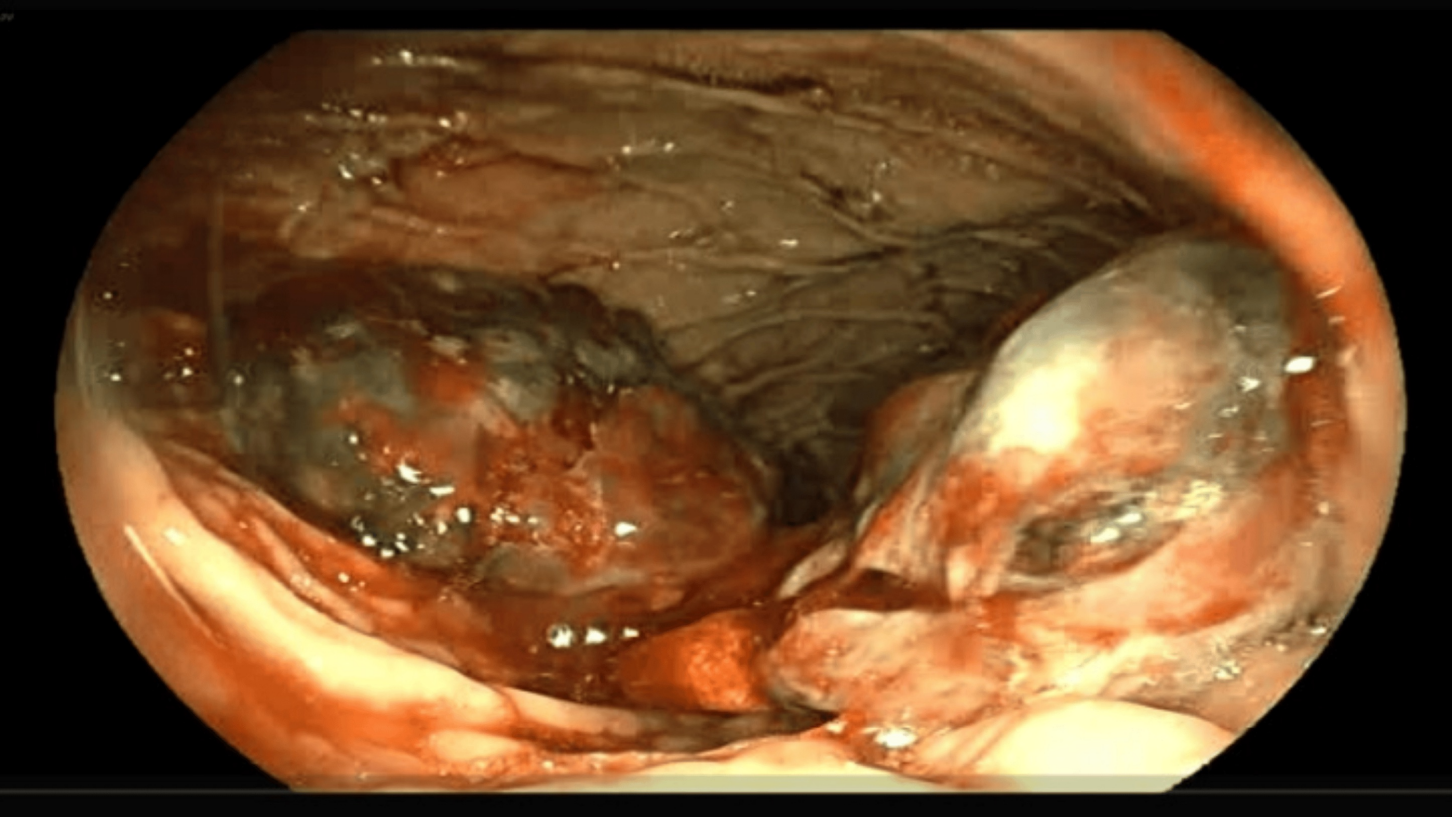
Endoscopic view of the tumor on the gastric body with active bleeding.

The known malignancy, which was primarily located in the gastric body, was identified during endoscopy. Active bleeding was observed from the tumor surface (Figure 2). The procedure was conducted with utmost care to minimize trauma to the friable tissue: CO_2_ inflation was used to reduce the risk of perforation, and no attempt was made to pass the pylorus, avoiding additional strain on the tumor. Given the diffuse nature of the bleeding and the substantial tumor area, a 10Fr catheter was selected to dispense the TC-325 powder, achieving hemostasis. In the immediate post-operative period, the patient complained of epigastric pain, which was initially attributed to the visceral distention associated with the gas propellant of the hemostatic device.

**Figure 3:**
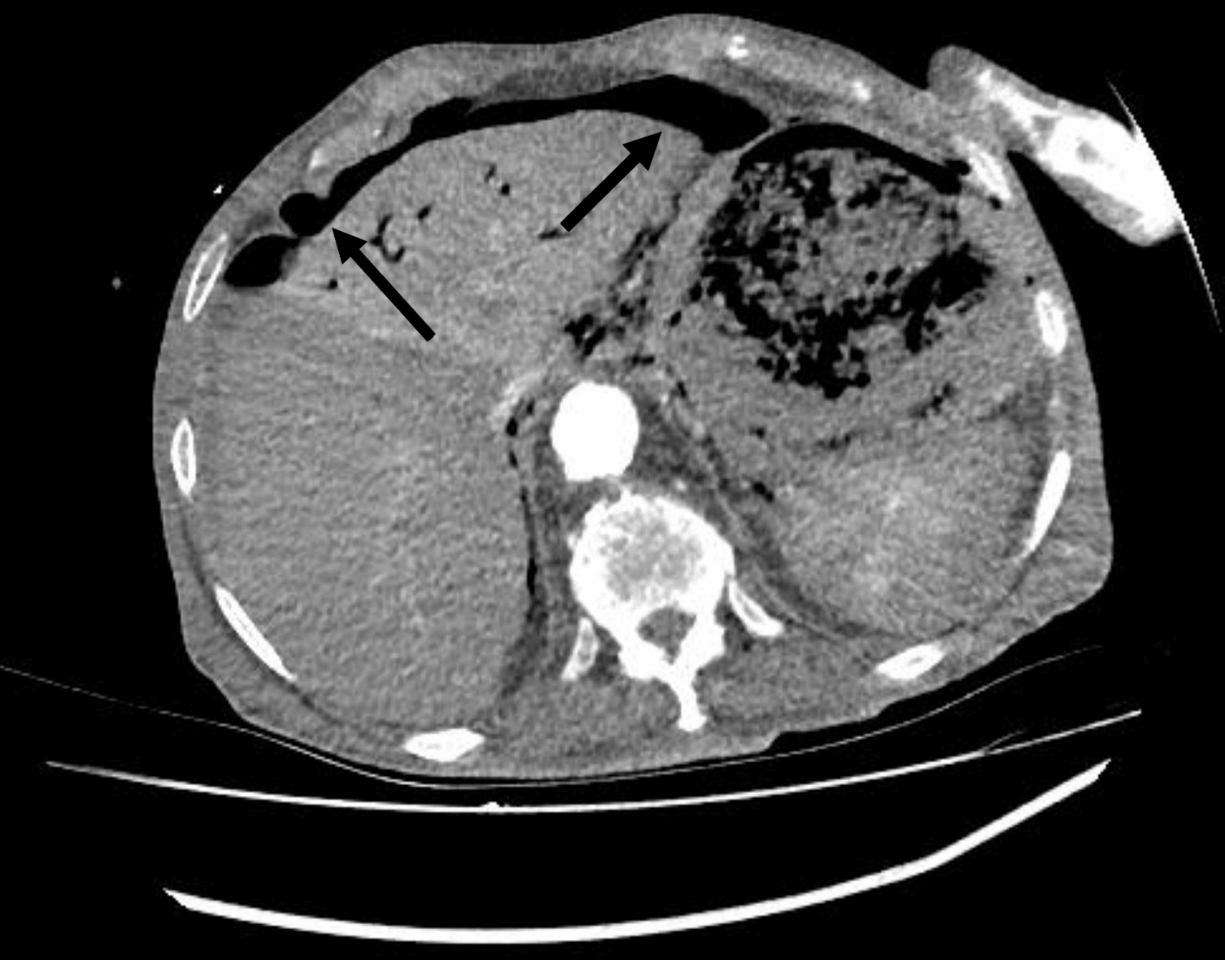
Presence of free air (arrows) and fluid in the peritoneal cavity after hemostasis with TC-325 powder. TC-325 (Hemospray; Winston-Salem, NC: Cook Medical)

Subsequently, the patient developed severe diffuse abdominal pain, fever, and leukocytosis. An urgent abdominal CT revealed free air and fluid in the peritoneal cavity, indicating visceral perforation (Figure 3). No extravasation of intravenous contrast into the bowel lumen was found. Due to the advanced stage of the disease and the high risk of surgical complications, the patient was not deemed a candidate for surgical intervention. Despite achieving initial hemostasis, the patient succumbed to complications two days later.

## Discussion

Endoscopy remains the cornerstone of treatment for patients presenting with NVUGIB, with resuscitation using IV fluids and blood transfusions being critical to stabilize patients prior to the procedure [1]. Current ESGE guidelines recommend endoscopy within 24 hours of admission, as urgent endoscopy (<12 hours) has not demonstrated superior outcomes compared to delayed intervention [5].

The use of Hemospray (TC-325) in similar cases has been associated with several beneficial effects, including significant reduction in transfusion requirements, improved quality of life, and long-term oncological outcomes and bridging to more definitive treatments, such as radiotherapy or surgery [6].

Recent studies have highlighted the efficacy of TC-325 in managing malignancy-related GI bleeding. A systematic review and meta-analysis by Chahal et al. demonstrated that TC-325 significantly improves hemostasis rates and reduces transfusion needs in patients with upper GI bleeding [7]. Similarly, Hussein et al. reported favorable outcomes in an international registry of patients treated with TC-325 for malignancy-related bleeding, with a high rate of initial hemostasis and low re-bleeding rates [6]. Karna et al. and Saeed et al. further supported these findings, showing that TC-325 is superior to standard endoscopic therapies in achieving hemostasis in malignancy-related bleeding [8,9].

However, as highlighted by Pittayanon et al., endoscopic treatment for patients with GI bleeding due to malignant tumors may sometimes be futile, particularly in the short-to-medium term [10]. Wang et al., in an editorial accompanying the Pittayanon study, noted that while some patients with tumor-related bleeding may be candidates for therapeutic endoscopic or surgical resection, many have advanced or incurable cancers. In these patients, TC-325 appears to provide improved short- to medium-term hemostasis compared to standard endoscopic therapy. For patients with end-stage malignancies, even a few weeks of palliative hemostasis can provide valuable time to consider palliative care options and manage their affairs before end-of-life [11].

Despite its benefits, the use of TC-325 is not without risks. In the present case, the patient experienced visceral perforation, a rare but serious complication. Advanced cancerous invasion of the gastric wall likely contributed to the friability of the tissue, increasing the risk of perforation. Clinicians must exercise caution when applying TC-325 in patients with advanced malignancies, particularly those with diffuse or long-standing disease. The forceful release of the hemostatic powder through the endoscope can cause tissue rupture and subsequent pneumoperitoneum [12].

The use of a 10Fr catheter for TC-325 application in this case may have contributed to the risk of perforation, given the friable nature of the tumor and the advanced stage of the disease. While there is no direct evidence comparing the perforation risk of 10Fr vs. 7Fr catheters in malignancy-related bleeding, the higher force of powder delivery with a 10Fr catheter could theoretically increase mechanical stress on weakened tissue. In high-risk cases, such as advanced malignancies, the use of a 7Fr catheter may be preferable to minimize trauma, despite potentially requiring more applications to achieve hemostasis. Future studies comparing catheter sizes in this context are needed to provide clearer guidance on optimal technique.

## Conclusions

TC-325 hemostatic powder is a valuable tool for managing NVUGIB in patients with malignant upper GI tumors, offering effective hemostasis in challenging cases. It significantly reduces transfusion requirements, improves quality of life, and serves as a bridge to more definitive treatments such as radiotherapy or surgery. However, visceral perforation, though rare, remains a serious complication, particularly in patients with advanced disease and friable tissue. Clinicians must remain vigilant for signs of perforation, as early recognition and intervention are critical to optimizing outcomes. This study underscores the need for caution when using TC-325 in high-risk populations and highlights the importance of balancing the benefits of hemostasis with the risks of complications in patients with advanced malignancies.
